# Enriched Air Nitrox Breathing Reduces Venous Gas Bubbles after Simulated SCUBA Diving: A Double-Blind Cross-Over Randomized Trial

**DOI:** 10.1371/journal.pone.0154761

**Published:** 2016-05-10

**Authors:** Vincent Souday, Nick J. Koning, Bruno Perez, Fabien Grelon, Alain Mercat, Christa Boer, Valérie Seegers, Peter Radermacher, Pierre Asfar

**Affiliations:** 1 Department of Medical Intensive Care and Hyperbaric Medicine, University Hospital, Angers, France; 2 INSERM U1083, CNRS UMR 6214, University Hospital, Angers, France; 3 Interactions Cellulaires et Applications Thérapeutique and DRCI Data management, SFR du pôle Santé; University Hospital, Angers, France; 4 Department of Anesthesiology. Institute for Cardiovascular Research, VU University Medical Center, Amsterdam, The Netherlands; 5 Institut für Anästhesiologische Pathophysiologie und Verfahrensentwicklung, Universitätsklinikum Ulm, Ulm, Germany; Charité—Universitätsmedizin Berlin, GERMANY

## Abstract

**Objective:**

To test the hypothesis whether enriched air nitrox (EAN) breathing during simulated diving reduces decompression stress when compared to compressed air breathing as assessed by intravascular bubble formation after decompression.

**Methods:**

Human volunteers underwent a first simulated dive breathing compressed air to include subjects prone to post-decompression venous gas bubbling. Twelve subjects prone to bubbling underwent a double-blind, randomized, cross-over trial including one simulated dive breathing compressed air, and one dive breathing EAN (36% O_2_) in a hyperbaric chamber, with identical diving profiles (28 msw for 55 minutes). Intravascular bubble formation was assessed after decompression using pulmonary artery pulsed Doppler.

**Results:**

Twelve subjects showing high bubble production were included for the cross-over trial, and all completed the experimental protocol. In the randomized protocol, EAN significantly reduced the bubble score at all time points (cumulative bubble scores: 1 [0–3.5] vs. 8 [4.5–10]; P < 0.001). Three decompression incidents, all presenting as cutaneous itching, occurred in the air versus zero in the EAN group (P = 0.217). Weak correlations were observed between bubble scores and age or body mass index, respectively.

**Conclusion:**

EAN breathing markedly reduces venous gas bubble emboli after decompression in volunteers selected for susceptibility for intravascular bubble formation. When using similar diving profiles and avoiding oxygen toxicity limits, EAN increases safety of diving as compared to compressed air breathing.

**Trial Registration:**

ISRCTN 31681480

## Introduction

Decompression sickness (DCS) may occur after compressed air breathing during open water Self Contained Underwater Breathing Apparatus (SCUBA) diving, even if empiric safety procedures are respected. During and/or after decompression, dissolved inert gas may convert to gas bubbles. Presence of gas bubbles precedes clinical symptoms [1]. It is generally accepted that the risk of DCS occurrence is related to the presence of intravascular bubbles [2], and their quantification using Doppler and/or ultrasound is a tool for studying decompression stress [3,4].

Increasing the fraction of inspired oxygen (FiO_2_) may reduce decompression stress by augmenting the so-called “oxygen window”, i.e. the diffusion gradient between the partial gas pressures into bubbles (high N_2_, low O_2_ tensions) and surrounding blood and/or tissues (low N_2_, high O_2_ tensions). Theoretically, higher blood and tissue oxygen partial pressures should lead to increased nitrogen dissolution and subsequent clearance from the body. Indeed, increasing the FiO_2_ from 0.4 to 1.0 during the ascent from dives, in which a rebreather circuit (closed circuit) was used, led to reduced intravascular bubble scores, demonstrating the relevance of the oxygen window during decompression [5]. Currently, in addition to reduced decompression times, gas mixtures with increased FiO_2_ (enriched air nitrox, EAN) are frequently used to improve diving safety. However, Thom *et al* reported that EAN with 32% of FiO_2_ did not affect intravascular bubble scores after a single open water SCUBA dive as compared to air breathing, when inspiratory N_2_ partial pressures were matched between groups [6]. Currently, no studies have evaluated the effect of EAN as compared to air breathing on bubble formation in similar diving profiles, and the suggested benefit of EAN on diving safety has therefore not been demonstrated. Therefore, we tested the hypothesis that EAN breathing reduces venous bubble scores and thereby the risk of DCS in volunteers selected for high post-decompression bubble formation as compared to air breathing. For this purpose, healthy volunteer divers underwent simulated dives in a hyperbaric chamber (HC) breathing air or EAN with similar diving profiles according to a double-blinded cross-over design.

## Material and Methods

### Subject characteristics

The Human Subjects Committee of the Centre Hospitalier Universitaire d'Angers approved this single center, prospective, double-blind study with crossover design. Since the current study was designed in 2001, the trial was registered retrospectively (ISRCTN 31681480). Written informed consent was obtained from all subjects. Forty-seven human volunteers were included. Inclusion criteria were age >18 years, diving experience as confirmed by possession of a French recreational diver license and absence of contraindication to dive. Exclusion criteria were: history of decompression accident(s), oxygen administration or diving within 24 hours before simulated dive, or absent or low intravascular bubble production (Bubble score ≤ 1; Table 1) after initial test decompression. A CONSORT Checklist of the current trial is presented in S1 File.

**Table 1:** Intravascular bubble score.

Grade 0	Complete absence of bubbles
Grade 1	Bubbles detected in less than 25% of cardiac cycles
Grade 2	Bubbles detected in 25% to 50% of cardiac cycles
Grade 3	Bubbles detected in more than 50% of cardiac cycles
Grade 4	Bubbles detected in all cardiac cycles

Intravascular bubbles scores assessed by pulmonary artery pulsed Doppler measurements, based on the score system as described by Spencer (18).

### Study protocol

All simulated dives took place in a hyperbaric chamber (HC) of Angers university hospital, France, between August 2002 and September 2007. The 47 divers underwent an initial simulated air dive to exclude subjects with low intravascular bubble production (Bubble score ≤ 1; Table 1) in order to select divers with high bubble production (Bubble score ≥ 2) and thereby maximize the possible effect size of our intervention. Twelve divers (10 men and two women) demonstrating high intravascular bubble production completed the study protocol in a randomized, double-blinded crossover setup. Group allocation occurred by one of the investigators who was not involved in data analysis. Block randomization was used per 4 included subjects. Divers were assigned to undergo one simulated dive while breathing air (Air; 21% O_2_) and one simulated dive breathing enriched air nitrox (EAN) with 36% O_2_ in a randomized order. EAN 36/64 was chosen to optimize the oxygen window while maintaining a near-maximal partial pressure of O_2_ at a depth of 28 meters of salt water (msw). Partial pressure of O_2_ in our study at maximal depth was 1.37 atm, whereas the advised maximum partial pressure of O_2_ is 1.6 atm to prevent neurologic oxygen toxicity and is usually set up at 1.4 atm for recreational SCUBA diving [7,8]. At least 24 hours delay between dives was required.

### Diving protocol

All divers were accompanied in the HC by a physician certified in hyperbaric medicine. During simulated dives, subjects breathed on a mouthpiece through a mechanical ventilator (Servo 900C, Siemens, Sweden) set on pressure support of 10 cmH_2_O to approach regular diving circumstances. To simulate pressurized air or EAN breathing conditions, FiO_2_ was set at 21% or 36%, respectively. The divers were blinded for the administered gas mixture during the dives and for the ultrasound recordings.

**Fig 1:**
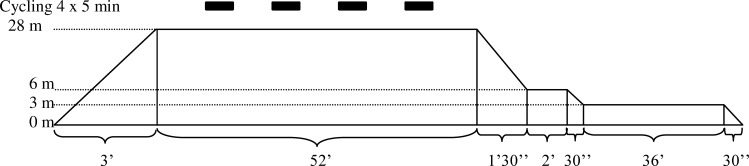
Simulated diving profile. Study compression profile as used for all simulated dives to a maximum depth of 28 msw, followed by two decompression steps at 6 msw and 3 msw. For further details see text.

The diving profile (Fig 1) was designed according to the decompression table of the French Navy and was used for all simulated study dives. After a descent at 10 m/min, divers remained for 55 minutes at a depth of 28 msw. Decompression was performed at a rate of 15 m/min until a first decompression stop of 2 minutes at 6 msw. Thereafter, decompression was continued at a rate of 6 m/min with an additional stop of 36 minutes at 3 msw. The bottom depth of 28 msw was chosen to maximize nitrogen saturation and decompression stress, yet still permitting EAN breathing. To simulate normal diving circumstances with effort and to facilitate nitrogen and oxygen uptake, subjects cycled at an energy expenditure of 50 Watts at 4 episodes of 5 minutes during bottom time on a stationary bicycle placed in the HC (Fig 1), with 5 minutes of rest between cycling. Temperature in the HC was maintained at 22–25° Celsius during stabilization of depths.

### Pulsed Doppler measurements

All subjects underwent pulsed Doppler measurements of the trunk of the pulmonary artery in order to quantify bubbles as primary outcome, in line with previous reports [9]. Measurements of one minute duration were performed and recorded by a certified cardiologist, blinded for the FiO_2_ at 0, 30, 60 and 90 minutes after decompression. Recordings were analyzed for bubble scores offline. Divers did not exercise between pulsed Doppler measurements.

### Bubble score calculation

A modified bubble score was determined over one minute of cardiac cycles, based on the Doppler score system originally derived by Spencer and modified by Eftedal and Brubakk (Table 1) [10,11]. Bubbles scores were analyzed independently by two blinded reviewers.

### Biometric measurements

Body height and weight were measured in all subjects. Body mass index (BMI) and body surface area (BSA) were determined using standard calculations. Additionally, body fat percentage by skinfold measurements according to Durnin and Womersley and waist-hip ratio were obtained [12].

### Trial registration

Retrospective registration was necessary since trial registration was uncommon at the time of design of the trial. The original study protocol and an English summary of the study protocol are presented in S2 and S3 Files, respectively.

### Statistical analysis

Data were analyzed using R software (R Foundation for Statistical Computing, Vienna, Austria), version 3.2.4 Each parameter was tested for normality with Kolmogorov-Smirnov tests and handled accordingly. All values are expressed as mean ± standard deviation or median with interquartile range [IQR] as appropriate. All included volunteers were considered as their own control subject since they each underwent one EAN and on Air dive. Since the bubble scores were calculated on an ordinal scale and given the size of analyzed sample, we used nonparametric tests for comparisons. The Higgins & Tashtoush formula for repeated measures (at 0, 30, 60 and 90 minutes after decompression) was used for the interaction alignments with the aligned Koch rank score (based on ranking the K² pairwise differences among the 4 levels of the repeated measures, regardless of treatment membership ranks), and the resulting p-value was reported [13,14] Between groups differences per time point and for the cumulative bubble score were analyzed using paired Wilcoxon tests adjusted for multiple comparisons by the Hochberg method [15]. Differences in biometric data were tested by Student’s T-test, Mann Whitney U tests or Chi-square tests, as appropriate. Correlations between bubble scores of the initial air dive and subject characteristics were evaluated with Pearson correlation coefficients. P < 0.05 was considered as statistically significant. An unplanned interim analysis was performed after inclusion of 12 divers, due to the impossibility of a power calculation before the study in the absence of previous data and due to a slowly advancing investigation because of complex logistics in a clinical hyperbaric chamber. After obtaining significant differences in primary outcome after inclusion of 12 divers, the seven remaining divers did not participate to the randomized dives.

## Results

### Subject characteristics

**Fig 2:**
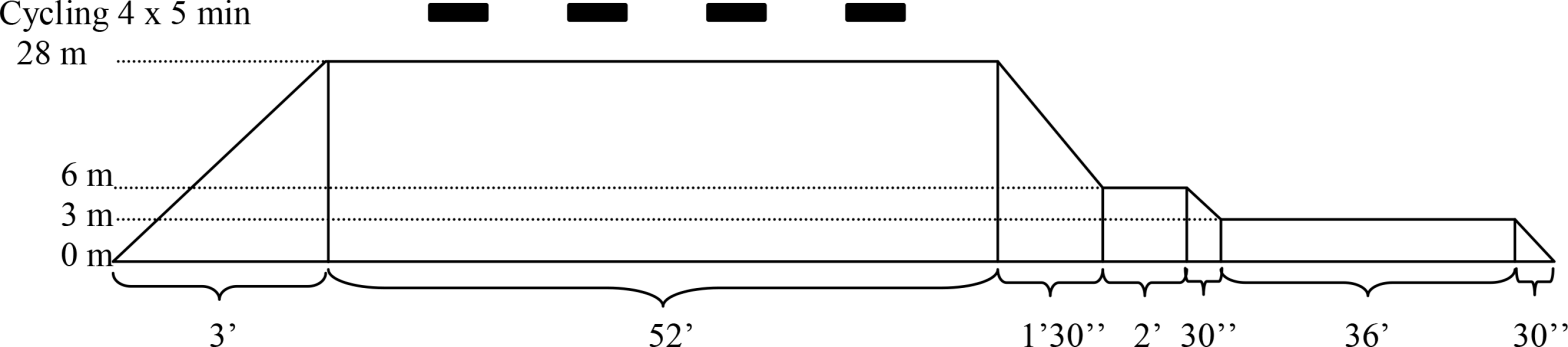
Inclusion and randomization flow diagram. EAN = Enriched air nitrox.

Out of the 47 included volunteers, 19 subjects were found to produce intravascular bubbles (Bubble score ≥2) upon decompression. The inclusion flow diagram is shown in Fig 2. Table 2 presents the characteristics of volunteers stratified according to the level of intravascular bubble production (Bubble score ≤1 versus ≥2) after the initial simulated dive breathing air. Increased bubble production was associated with higher age. No differences in biometric data were observed between subjects demonstrating high or low levels of venous gas emboli.

**Table 2:** Subject characteristics.

	Bubble score ≥ 2 (n = 19)	Bubble score ≤1 (n = 28)	P-value
Age (years)	45 ± 9	39 ± 9	0.02
Gender (M/F)	14 / 5	21 / 7	1.0
BMI (kg/m^2^)	25 ± 4	24 ± 2	0.09
BSA (m^2^)	1.9 ± 0.2	1.9 ± 0.2	0.36
Body fat (%)	22.6 ± 5.0	21.1 ± 7.0	0.43
Waist/hip ratio	0.87 ± 0.08	0.83 ± 0.07	0.14
CMAS diving level (Autonomous diver level 1/2/3/4/ instructor)	2/4/4/2/7	4/5/8/3/8	0.63

Subject characteristics of subjects exhibiting high bubble score ≥ 2 or low Bubble score ≤1; n, number of subjects in each group. Values are means ± SD. Data was tested by Student’s T-test, Mann Whitney U tests or Chi-square tests, as appropriate. BMI = body mass index, BSA = body surface area, CMAS = Confédération Mondiale des Activités Subaquatiques or World Underwater Federation.

### Inter-rater agreement

All intravascular bubble scores were determined independently by two investigators blinded for group assignment. Inter-rater agreement was excellent with a Kappa level of 0.915 (P < 0.001).

### Intravascular bubble analysis

**Fig 3:**
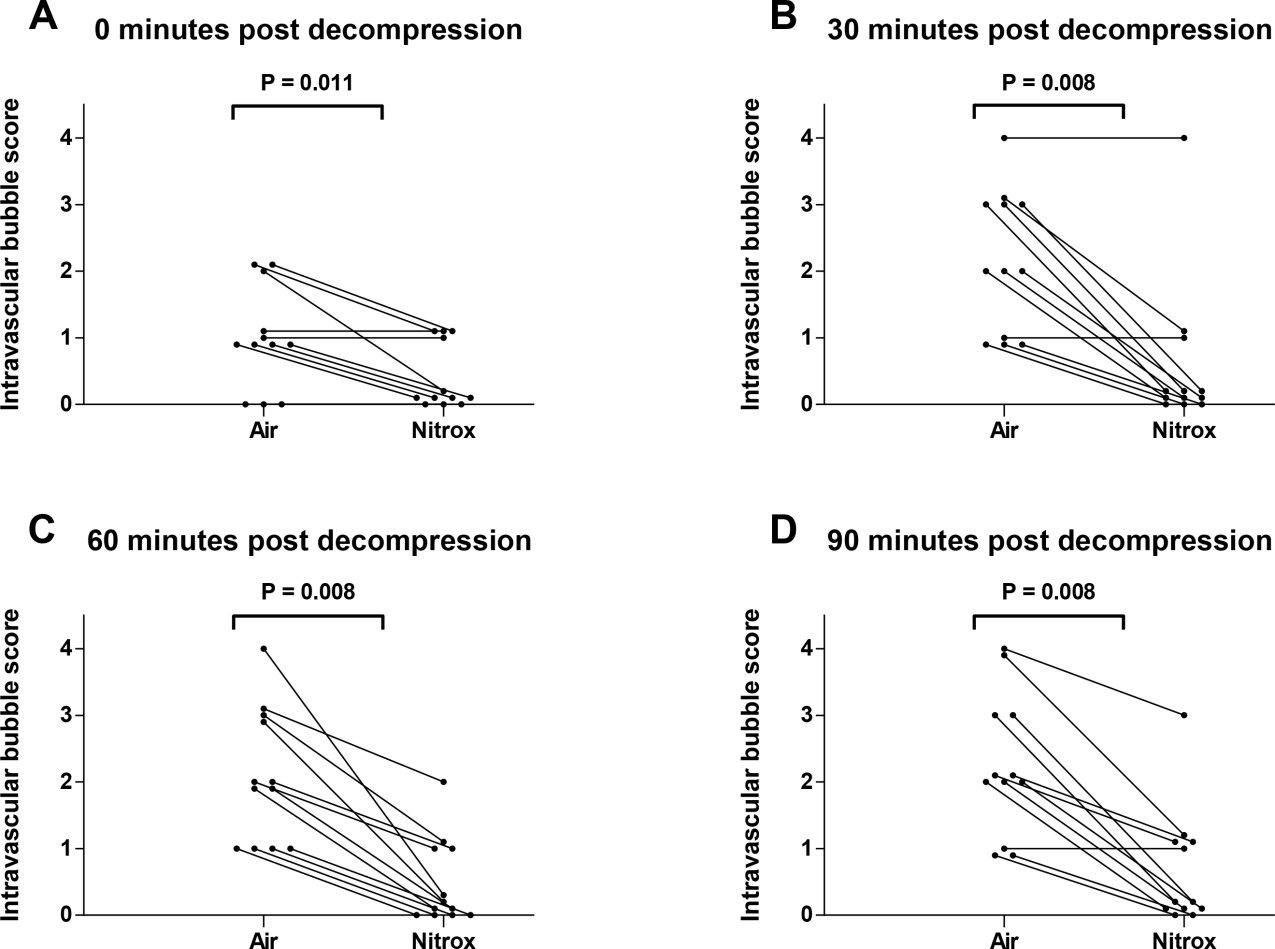
Intravascular bubble scores. Intravascular bubble scores at T0 (Panel A), T30 (Panel B), T60 (Panel C) and T90 (Panel D) for all individuals undergoing both air and enriched air nitrox dives. All panels were tested with Wilcoxon tests adjusted for multiple comparisons by the Hochberg method.

**Fig 4:**
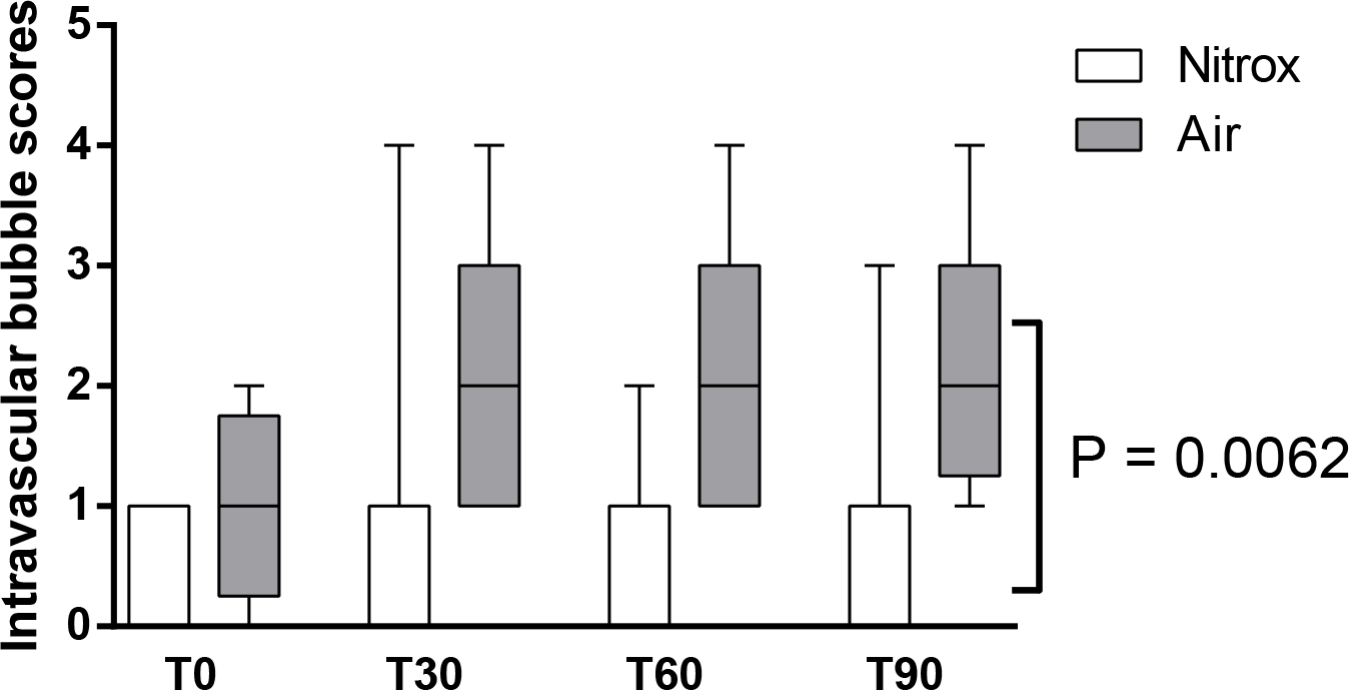
Grouped intravascular bubble scores. Median, interquartile ranges and ranges are presented for intravascular bubble scores in both groups. Scores were tested with Koch rank test for between group effect over time.

Fig 3 shows the individual pulmonary artery bubble scores for the randomized dives immediately (Panel A) as well as 30 (Panel B), 60 minutes (Panel C), and 90 minutes (Panel D) post decompression. EAN breathing markedly reduced the number of bubbles at all time points, the maximum difference being present at 30 (0 [0–1] vs. 2 [1–3]; P = 0.008), 60 (0 [0–1] vs. 2 [1–3]; P = 0.008), and 90 minutes post-decompression (0 [0–1] vs. 2 [1.25–3]; P = 0.008). Consequently, EAN lowered the total bubble load following decompression as assessed by the sum of the four consecutive bubble scores (1 [0–3.25] vs. 8 [4.75–9.5]; P = 0.008). A Koch rank test for grouped and repeated measures over the four time points showed a significant difference between groups (P = 0.0062; Fig 4).

Table 3 shows the results of the bubble quantification between the two air dives for the 12 included subjects (initial air dive versus randomized air dive). At 30 and 60 minutes after decompression, bubble scores were lower after the second air dive than after the first air dive. No further acclimatization effect was detected as evidenced by an absence of a reduction in cumulative bubble scores between initial air and randomized air dives for subjects starting performing the air dive as second or as third dive (2.5 [0.5–5] versus 2.5 [1–5]).

**Table 3:** Intravascular bubble scores of two air dives.

	Initial air dive	Randomized air dive
T 0	1 [1–3]	1 [0.25–1.75]
T 30	3 [2–4]	2 [1–3]
T 60	3 [2–3]	2 [1–3]
T 90	3 [2–4]	2 [1.25–3]

Bubble scores for subjects (n = 12) undergoing both initial air dive and a randomized study air dive. Data are presented as median and interquartile ranges.

### Decompression incidents

Three minor DCS incidents occurred during the cross-over study protocol. None of the twelve EAN dives was associated with any incident, all three incidents took in the air dives (P = 0.217 between groups; N = 24 dives). Another eleven DCS incidents were observed in the 47 initial air dives. All DCS incidents presented as cutaneous itching during the ascent (3 events) or within one hour post decompression (11 events) without concomitant objective clinical signs. Dives leading to DCS incidents were not associated with an increase in the sum of the bubble scores (DCS incident: 3 [1–7.75], N = 14 vs. no DCS incident: 4.5 [1–11], N = 57; P = 0.344), two incidents even occurred in subjects with a total bubble score of 0.

### Correlations of decompression stress with biometric data

There was a weak relationship between the sum of the intravascular bubble score and age (Pearson r = 0.33, P = 0.021), but not with BMI or body fat percentage. Subjects with decompression incidents had lower waist/hip ratios (0.77 ± 0.05 vs. 0.90 ± 0.04; P = 0.001), higher body fat percentages (27.1 ± 4.8% vs. 19.5 ± 3.6%; P = 0.015), and a tendency towards lower a BSA (2.0 ± 0.1 m^2^ vs. 1.8 ± 0.2 m^2^; P = 0.075).

## Discussion

To the best of our knowledge this study is the first to demonstrate that EAN reduces venous gas bubble formation as compared to air breathing during simulated SCUBA diving in volunteers selected for susceptibility for bubble formation. Moreover, EAN prevented the incidence of decompression stress. Except for weak correlations with BMI and age, bubble scores were not related to any other demographic parameter.

At first glance, our data of an EAN-induced reduction of venous bubble formation are in contrast with Thom *et al* reporting that EAN (FiO_2_ 32%) breathing during wet dives at 22.5 msw did not affect the maximum bubble score when compared to compressed air breathing [6]. However, these authors compared EAN breathing at 22.5 msw to compressed air at 18 msw in order to achieve matched inspired N_2_ partial pressures. Hence, any oxygen window effect of EAN versus compressed air breathing was less pronounced (inspired O_2_ pressures of 1.04 vs. 0.59 atm and N_2_ partial pressures of 2.21 vs. 2.21 atm, respectively) than in our study (inspired O_2_ pressures of 1.37 atm vs. 0.79 and inspired N_2_ partial pressures of 2.43 vs. 3 atm). Thus, our findings are in good agreement with important role of the O_2_ suggested by Blatteau et al: increasing the FiO_2_ from 0.4 to 1.0 at 10 msw during the final part of the decompression from a simulated dry chamber dive markedly reduces venous bubble formation [5]. However, breathing a single gas mixture during an entire dive profile is more practical for divers than changing gas mixtures during ascent. Another study evaluated the effect of air versus EAN on post-diving fatigue following simulated dry dives [16]. No difference in fatigue between groups was detected, although fatigue is an unspecific symptom of DCS [16]. It is unlikely that the lacking preselection of subjects prone to intravascular bubble formation prevented the demonstration of a possible advantage of EAN in the study of Thom et al, as the bubble formation in their study was high with a mean bubble score of 3 at 95 minutes after the dive [6]. However, it should be noted that Thom *et al* investigation had different endpoints than the current investigation, did not have a double-blind randomized design as in our study, and was designed to match inspiratory N_2_ partial pressures between groups [6]. These differences may explain the absence of reduction in bubble formation rate by EAN breathing during diving in the study by Thom *et al* [6].

Several recent studies have shown that the pathophysiology of DCS is more complex than decavitation of inert gases, and additionally includes microparticle formation, neutrophil activation and endothelial dysfunction [6,17,18]. Although associations have been detected of DCS with microparticles and blood-endothelial interactions, it remains to be elucidated whether the observed processes are cause or consequence of DCS [6,18]. Indeed, in the current study two subjects developed symptoms of DCS without having bubbles detected by pulmonary artery pulsed Doppler measurements, suggesting that intravascular bubbles are not solely explanatory for DCS symptoms.

The cross-over study protocol allowed for the subjects to serve as their own control and thus minimized bias due to between-subject variability. Bubble formation was lower after the second than the first simulated air dive, in accordance with a previous observation of Zanchi *et al* in wet dives [19]. We can only speculate on this finding, but “acclimatization or learning” to simulated dry chamber dives might have assumed importance in this context: e.g., reduced emotional stress resulting in attenuation of adrenergic stimulation and, consequently, lower cardiac output would attenuate tissue nitrogen uptake, whereas improved fluid intake would increase both physical N_2_ dissolution and elimination. Regardless of the explanation, our data demonstrate the possible presence of a time-dependency of the bubble formation, resulting in poor intra-individual reproducibility of the diving effects. Therefore, the randomized cross-over protocol proved to be mandatory to discriminate differences of the two gas mixtures on venous bubble formation independent of within subject variability.

Our diving schedule was chosen to provoke N_2_ bubble formation and possibly DCS symptoms. In fact, both the number of DCS incidents (20% of simulated dives) and the number of subjects with high bubble scores (40% of study subjects) were high. Nevertheless, all decompression incidents were minor, and consisted of cutaneous itching (no cutis marmorata or any other objective erythema). Even with a maximal O_2_ partial pressure of 1.37 atm, our diving schedule was still under below the accepted threshold of O_2_ toxicity threshold and we did not observe and sign of O_2_ toxicity [7].

There was a weak correlation between the total bubble score and age, whereas bubble production was not directly related to body fat percentage or BMI. Previous reports also demonstrated increased bubble formation associated with older divers (above 30–40 years old) [20,21]. Equivocal data are available on the role of both body mass and fat: both no influence of body fat or reduced bubble grading were reported in leaner volunteers [21–23]. Although our study was not powered to detect a relation between bubble production and body fat percentage, we think that our observation of a lacking relation between these variable well agrees with the existing literature: the latter study by Carturan *et al* did not control for physical fitness between leaner and fatter subjects, and thus any reduced bubble formation in the leaner divers was most likely due to higher fitness in these subjects [21].

The commonly accepted hypothesis on bubble generation is that N_2_ supersaturates in particular in fat tissue, due to its higher solubility in lipid than in aqueous solutions. It remains unknown, if number of venous emboli is a valid outcome parameter for N_2_ saturation of fat tissue. This question is underscored in our study by the discrepancy between the relation of demographic parameters and either bubble scoring or DCS symptoms: while there was no relation between body fat and intravascular bubbles, the presence of DCS symptoms was associated with higher body fat percentages. In fact, 81% of fatal diving accidents were reported to occur in divers with BMI ≥ 25 [24].

A limitation of this study includes the use of a HC instead of non-simulated dives. Conversely, circumstances could be optimally controlled, which is important for the current proof-of-concept and results should be reproduced for wet dives [25].

In conclusion, our results provide evidence for increased safety of EAN breathing during SCUBA diving as compared to compressed air breathing, when using identical diving profiles and not exceeding oxygen toxicity limits. EAN gas is widely available and these results could therefore confirm immediate utility to reduce DCS risks.

## Supporting Information

S1 File CONSORT Checklist. (DOC)

S2 File Original study protocol in French. (PDF)

S3 File English summary of study protocol. (DOC)
