# Correction: Enriched Air Nitrox Breathing Reduces Venous Gas Bubbles after Simulated SCUBA Diving: A Double-Blind Cross-Over Randomized Trial

**DOI:** 10.1371/journal.pone.0165771

**Published:** 2016-10-26

**Authors:** Vincent Souday, Nick J. Koning, Bruno Perez, Fabien Grelon, Alain Mercat, Christa Boer, Valérie Seegers, Peter Radermacher, Pierre Asfar

Fig 1 is incorrectly duplicated as Fig 2. Please view the correct Fig 2 here.

**Fig 2:**
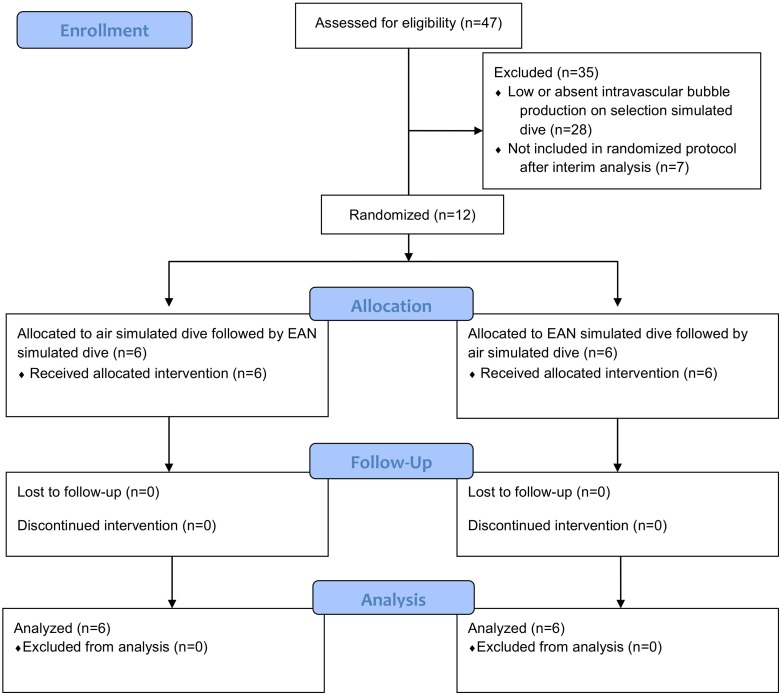
Inclusion and randomization flow diagram. EAN = Enriched air nitrox.
