# Supplementary material for: Enriched Air Nitrox Breathing Reduces Venous Gas Bubbles after Simulated SCUBA Diving: A Double-Blind Cross-Over Randomized Trial
Source: PLoS One. 2016 May 10;11(5):e0154761. doi: 10.1371/journal.pone.0154761 (PMC4862661; doi:10.1371/journal.pone.0154761)
Supplement: S2 File — (PDF) [file pone.0154761.s002.pdf]

Service de Réanimation Médicale – Unité d'Oxygénothérapie Hyperbare

---

COMPARAISON PAR ECHOCARDIOGRAPHIE DOPPLER DU PHENOMENE BULLEUX A L'ISSUE D'UNE PLONGEE A L'AIR ET AU NITROX

Février 2001

Investigateur principal : Dr Pierre Asfar (1)

Co-investigateurs : Bruno Perez (Médecin généraliste non thésé, diplômé de Médecine Hyperbare et subaquatique)
Dr Vincent Souday (1)
Pr Philippe Alquier (2)

Promoteur : CHU ANGERS

Type d'étude : Etude randomisée en double aveugle

(1) Praticien Hospitalier, Service de Réanimation Médicale, CHU, 4 rue Larrey 49033 Angers Cedex 01.
(2) PU-PH, Service de Réanimation Médicale, CHU Angers.

I) INTRODUCTION

L'**air comprimé** est le gaz respiratoire le plus utilisé en plongée de loisir depuis une cinquantaine d'années. Il est composé de 21% d'oxygène et de 79% d'azote et d'un infime pourcentage de gaz rares et inertes (hélium, hydrogène, argon…). L'**oxygène** est toxique pour le système nerveux central de l'homme s'il est respiré à une pression partielle de 1.7 bars (risque de crise comitiale). Cela interdit l'utilisation de l'air comprimé au-delà de 70 mètres, profondeur à partir de laquelle la pression partielle d'oxygène de 1.7 bars est atteinte. Il est aussi toxique pour les poumons (réaction inflammatoire) s'il est respiré à une pression partielle supérieure à 0.5 bar pendant plusieurs heures. En pratique, les temps d'exposition à l'oxygène nécessaires pour révéler la toxicité pulmonaire ne sont jamais rencontrés en plongée autonome de loisir (PAL). L'autre gaz majoritaire dans l'air est l'**azote** ; il est classiquement admis que ce seul gaz soit pris en compte quand il s'agit de saturation de l'organisme en gaz inertes.

Toutes les plongées à l'air, quels que soient leurs paramètres de temps et de profondeur, sont à l'origine de bulles d'azote en fin de remontée et lors du retour en surface générant un risque d'**accident de décompression** (ADD). L'importance du phénomène bulleux dépend de la **masse d'azote dissoute dans l'organisme** (liée à la durée et à la profondeur de la plongée) et du **profil de décompression** (PD) choisi par le plongeur pour assurer sa désaturation.

Un mélange suroxygéné connu depuis un siècle et couramment utilisé par les différentes marines nationales depuis cinquante ans commence à concurrencer l'air comprimé pour un usage de loisir. Il s'agit du **Nitrox**. Ce mélange associe comme l'air, l'azote et l'oxygène, mais le pourcentage de ce dernier est supérieur à 21% (il peut-être de 32, 36, 40 voire 50 et 70%). Comme il contient plus d'oxygène sa profondeur maximale d'utilisation sera impérativement inférieure à 70 mètres. Son usage implique donc un risque de toxicité neurologique plus important qu'avec l'air comprimé ; on parle classiquement de **risque hyperoxique**. Par ailleurs, le Nitrox est moins riche en azote que l'air ; la masse d'azote dissoute dans l'organisme du plongeur sera donc diminuée. Par conséquent, on peut espérer une **diminution de la saturation en cours de plongée et une diminution du phénomène bulleux à son issue**; ce qui justifie son actuel engouement.

En pratique, la plongée au Nitrox soulève deux questions : celle du **risque hyperoxique** et celle de la **désaturation.**

Le risque hyperoxique intéresse avant tout le plongeur qui va respirer le mélange suroxygéné. Actuellement, ce risque est assez bien maîtrisé. De nombreuses études ont permis d'évaluer le seuil de toxicité neurologique et pulmonaire d'un mélange suroxygéné en fonction de la profondeur à laquelle il était respiré et du temps pendant lequel il était respiré (4, 13, 14, 15, 12). Il en découle des codes pratiques d'usage des Nitrox accessibles lors de formation tout public. Le risque hyperoxique intéresse aussi le technicien Nitrox d'un club qui aura en charge la fabrication du mélange. Ce dernier devra manipuler de l'oxygène pur en tenant compte de son inflammabilité au contact des graisses et contrôler rigoureusement le pourcentage d'oxygène du mélange avant que le plongeur ne le respire.

Qu'en est-il de la désaturation à l'issue d'une plongée au Nitrox ?

Depuis presque un siècle, les plongeurs à l'air comprimé utilisent des tables qui donnent le PD (durée et profondeur des paliers à effectuer) en fonction des paramètres temps et profondeur de la plongée. Ces tables qui ont bénéficié de nombreux perfectionnements permettent actuellement de gérer au mieux la désaturation d'un plongeur ayant respiré un mélange contenant 79% d'azote. L'oxygène restant n'est classiquement pas pris en compte dans les phénomènes de saturation et dans la genèse du phénomène bulleux du fait de son utilisation cellulaire. Dès lors, les PD reconnus pour l'AC sont théoriquement utilisables pour gérer la désaturation d'un plongeur ayant respiré un mélange appauvri en azote. C'est le cas des mélanges suroxygénés respirés en PAL ; ces Nitrox saturant théoriquement moins l'organisme que l'AC . L'application des PD prévus pour l'AC permettrait donc aussi une marge de sécurité supplémentaire vis à vis du risque d'ADD. Cette position est partagée par la plupart des hyperbaristes (5,6) et les écoles de plongée l'enseignent (1,21). Certains discutent même l'usage des Nitrox chez des sujets supposés à risque tels que ceux ayant une prédisposition plus marquée au phénomène bulleux (selon l'âge, le poids, leur consommation d'oxygène à l'effort et leur entraînement physique) et/ou ceux rencontrant des conditions de plongée prédisposantes (froid, effort avant et après la plongée, fatigue).(3,5,10)

Les Nitrox sont donc des mélanges suroxygénés, théoriquement moins saturant que l'AC. Leur usage en PAL associé à celui des tables de décompression à l'AC permettrait des plongées plus sûres vis à vis du risque d'ADD. Ce dernier avantage communément admis est très difficile à mettre en évidence sur le plan statistique du fait de la faible incidence des ADD lors des plongées à l'AC (8). Il n'a cependant pas été évalué sur le plan physiologique avec les moyens d'investigation ultrasonographiques dont nous disposons actuellement. Par ailleurs, cet avantage n'existe qu'au prix d'une suroxygénation du mélange respiré. Bien que le risque hyperoxique soit connu, il est caractérisé par une importante variabilité inter et intra individuelle faisant redouter sa survenue à l'occasion d'une large vulgarisation des Nitrox.

II) OBJECTIF DE L'ETUDE

Dans le domaine de la plongée commerciale, la tendance actuelle est à la vulgarisation des Nitrox. Ces mélanges respiratoires suroxygénés sont supposés plus sûr que l'AC vis à vis du risque d'ADD mais sont potentiellement plus toxiques du fait de leur taux élevé d'oxygène. Dans ce contexte particulier, il nous paraît légitime de disposer d'arguments forts en faveur de leur principal avantage : la diminution du risque d'ADD. Le phénomène bulleux intravasculaire généré par la désaturation des tissus à l'issue d'une plongée peut être évalué par des méthodes ultrasongraphiques non invasives (16,17). L'importance du phénomène bulleux s'avère par ailleurs corrélé au risque d'ADD(11).

Nous nous proposons donc d'étudier le phénomène bulleux détecté par échodoppler cardiaque à l'issue d'une plongée à L'AC chez un plongeur susceptible d'expérimenter un phénomène bulleux détectable et de le comparer à celui détecté à l'issue d'une plongée au Nitrox (paramètres et PD identiques à la plongée à l'AC) chez le même sujet.

La question à laquelle nous allons tenter de répondre est donc :

"en comparaison avec l'air comprimé, le nitrox diminue-t-il le phénomène bulleux à l'issue d'une plongée dont le PD est dicté par une table à l'air ?"

III) METHODES

1- SCHEMA GENERAL DE L'ETUDE

➢ Etude contrôlée en double aveugle et en cross-over

- Concernant l'intensité du phénomène bulleux, les plongeurs se répartissent classiquement en « forts bulleurs », « moyens bulleurs » et « faibles bulleurs » (16). Afin de mettre en évidence une diminution du phénomène bulleux entre l'AC et le Nitrox, nous utiliserons une sélection de plongeurs appartenant aux « forts bulleurs » et « moyens bulleurs ». A cette fin, les sujets bénévoles seront conviés à réaliser une plongée fictive à l'AC en caisson (paramètres : 55 minutes à 28 mètres) et bénéficieront d'une analyse échocardiographique et doppler du phénomène bulleux. Les sujets présentant un indice de Kisman supérieur ou égal à 1 seront sélectionnés. En pratique, chaque "Plongée Test" regroupera 4 à 5 sujets dans le caisson. Une huitaine de "Plongée Test" devrait nous permettre de recruter 18 sujets bulleurs.

- Dès que le sujet aura rempli les critères d'inclusion (en l'absence de critères d'exclusion), il sera **randomisé** dans l'un des deux groupes suivants : **A** et **B**.

Les sujets de chaque groupe feront deux « plongées » différentes :

- une plongée à l'Air Comprimé dont le PD sera déterminé à partir de la table à l'air comprimé MN 90 (« *plongée Air* »);
- une plongée au Nitrox dont le PD sera identique au précédent (« *plongée Nitrox* »);

Pour les plongeurs du **groupe A** l'ordre des « plongées » sera :

- « *plongée Air* »
- « *plongée Nitrox* »

Pour les plongeurs du **groupe B** l'ordre des « plongées » sera :

- « *plongée Nitrox* »
- « *plongée Air* »

- **Le sujet ne connaîtra pas le mélange respiré.**
- **Chaque sujet est son propre témoin.**
- A l'issue de chaque plongée, le Dr Asfar réalisera une détection bullaire par échodoppler. Le principal critère de jugement sera le débit bullaire cardiaque au niveau du cœur droit avant le passage des bulles dans le filtre pulmonaire (cf. critères de jugement). Le médecin qui réalisera les mesures échodoppler à la sortie du caisson **ne connaîtra pas le mélange respiré qui vient d'être testé.**

➢ Population

Il est prévu d'inclure **18 sujets**. Ils seront répartis dans les deux groupes A et B qui comporteront donc 9 sujets chacun.

➢ Critères d'inclusion :

Tous les critères suivants doivent être présents :

- Sujet volontaire.
- Age supérieur ou égal à 18 ans.
- Les sujets doivent être plongeurs.
- Absence de contre-indication temporaire à la plongée : ORL (otites, rhinopharyngites, traumatisme du tympan...), pulmonaires (asthme, pathologies infectieuses ou inflammatoires), cardiaques (HTA sévère, valvulopathies, péricardite), gynécologiques (grossesse), psychiatriques (traitement antidépresseur, anxiolytique ou neuroleptique), stomatologiques (caries, prothèses amovibles), toute chirurgie récente et prise de médicaments.
- Nous choisissons d'inclure dans l'étude les sujets présentant un phénomène bulleux détectable lors d'une plongée test à l'AC dont les paramètres et le PD seront ceux du protocole. Le critère d'inclusion choisi est un indice de sévérité de Kisman supérieur ou égale à 1.

➢ Critères d'exclusion :

- ADD de type II récent ou non.
- Age inférieur à 18 ans.
- Grossesse.
- Oxygénothérapie ou plongée datant de moins de 24 heures.

NB : Tous les sujets participants à l'étude sont des plongeurs licenciés FFESSM. Comme leur appartenance à un club de plongée et donc l'obtention de leur licence FFESSM sont conditionnés par le passage d'un examen de non contre indication à la plongée subaquatique, les sujets sont aptes à être comprimés dans un caisson hyperbare. Concernant maintenant l'aptitude à la plongée avec mélanges suroxygénés, il n'existe actuellement pas de consensus sur la nature particulière de l'examen clinique et des examens complémentaires afin d'établir l'aptitude. En particulier, le test de sélection en caisson avec exposition à une pression partielle d'oxygène de 2.8 ATA n'est actuellement plus utilisé par les médecins militaires car sa valeur prédictive est insuffisante. D'autre part, le tracé EEG n'apporte pas un critère nécessaire et suffisant pour détecter une hypersensibilité à l'oxygène chez un individu. L'expérience montre qu'il n'existe pas de corrélation entre le tracé et la survenue d'une crise hyperoxique (9). Cependant, on sait que les deux organes cibles intéressés par la toxicité de l'oxygène sont le système nerveux central et les poumons. Aussi, grâce à l'interrogatoire, le questionnaire et l'examen clinique (auscultation pulmonaire, peak flow) précédant l'expérimentation, nous nous attacherons à éliminer les éventuels sujets à risque.

- <u>Organigramme des étapes de l'étude</u>

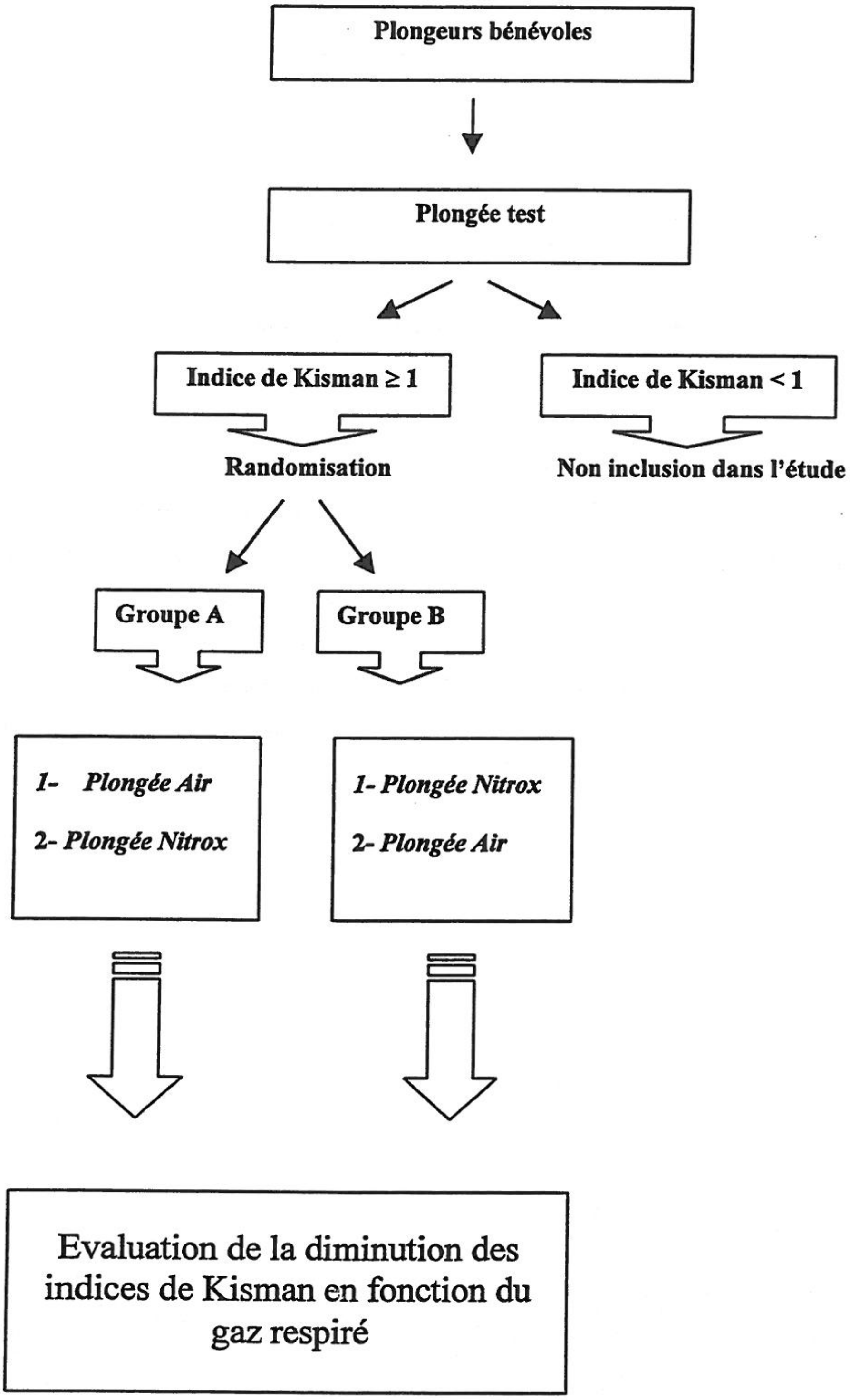

2- DEROULEMPENT DU PROTOCOLE

21- Calendrier de l'étude

L'étude prévoit deux plongées différentes pour chaque sujet. Un sujet sera comprimé à chaque séance dans un des compartiments du caisson. Trente-six séances de caisson doivent donc être programmées. Si la disponibilité des plongeurs nous permet de réaliser deux séances par semaine, l'étude durera approximativement quatre mois et demi.

22- Attribution de la plongée

Pour tous les sujets inclus dans l'étude, l'attribution de la plongée se fera par ouverture d'une enveloppe numérotée obtenue à partir d'une table de randomisation (équilibrée par bloc de neuf).

23- Déroulement des plongées

- Toutes les plongées se feront « au sec » en caisson hyperbare. Chaque séance permettra de comprimer un plongeur. Il y aura un plongeur dans un des deux compartiments du caisson. En cas d'incident (crise hyperoxique), le plongeur sera décomprimé avec l'accompagnant. Si un patient du service de réanimation (urgences de réanimation médicale) nécessite une séance urgente d'oxygénothérapie hyperbare, l'autre compartiment reste disponible.

- Les sujets respireront l'air comprimé ou le Nitrox sur un respirateur en mode « aide inspiratoire » (à 10 cm d'eau) au moyen d'un embout buccal type "deuxième étage d'un détendeur de plongée classique" adapté au tuyau d'admission-évacuation du respirateur (la programmation de l'appareil devra être dissimulée). Ce procédé devrait reproduire assez fidèlement les résistances rencontrées lors de l'usage d'un détendeur classique de plongée. Le sujet portera par ailleurs sur le visage un masque de plongée afin d'éviter toute inspiration nasale de l'ambiance du caisson.

- Pendant la plongée, le sujet devra se soumettre à quelques exercices physiques afin de reproduire les phases d'activité et de repos d'une plongée de loisir « normale ». Quatre séances de pédalage sur un ergocycle de cinq minutes chacune sont prévues pendant la plongée. L'effort fourni devra être de 50 Watts (cf. cahier de protocole) . L'effort sera standardisé grâce à un contrôle electrocardiographique (le rythme cardiaque du sujet devant se stabiliser dans un intervalle compris entre 110 et 130 pulsations minutes).

- Enfin, un médecin hyperbariste accompagnera les sujets lors de chaque séance. Il surveillera les phases de compression et décompression, gérera le réglage des respirateurs, donnera le « timing » des efforts et surveillera pouls et tension.

Envisageons maintenant les deux plongées proposées à chaque sujet.

➢ **Plongée à l 'air comprimé (*Plongée Air*) :**

Elle sera considérée comme la **plongée de référence** pour quantifier le phénomène bulleux. L'intensité du phénomène bulleux à l'issue de cette plongée sera comparée à celui constaté à l'issue de la plongée au Nitrox.

55 minutes à 28 mètres (3.8 bars) ; gaz respiré = Air Comprimé ; plongée de type " carrée ".

Le terme « carré » signifie qu'une fois que la profondeur maximale est atteinte, les sujets y seront maintenus jusqu'à ce que la décompression soit entreprise. Le schéma ci-dessous représente le profil de la *PLONGEE Air*.

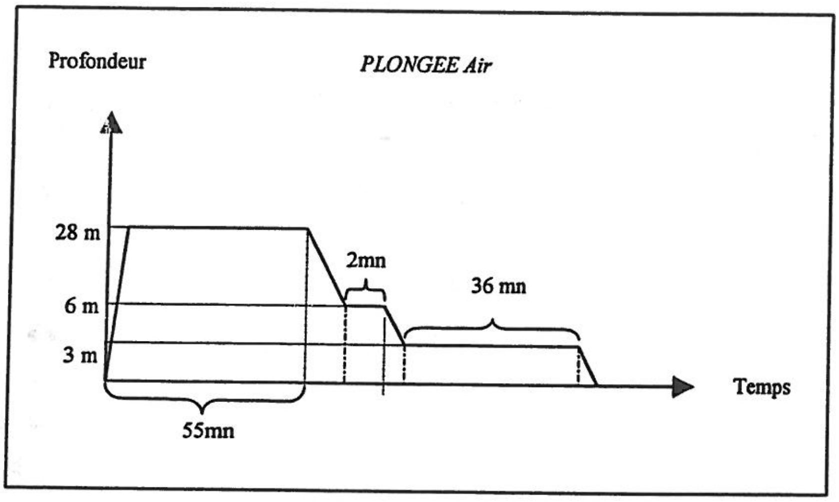

Temps de compression : Pour rester proche des conditions réelles de plongée, la compression sera réalisée en 3 minutes. Ce temps de compression sera compris dans les 55 minutes pour déterminer le PD à partir de la table de référence, comme il est pratiqué en plongée sportive.

Table de référence : MN 90 (table de référence pour la fédération française d'études et sports sous-marins [FFESSM], la plus utilisée en France par les plongeurs loisir utilisant des tables – cf. annexe 1). Le PD sera donc déterminé à partir de cette table.

Profil de Décompression (suivant la table MN 90) :

Paliers à réaliser : 2 minutes à 6 mètres
36 minutes à 3 mètres

Temps de remontée de 28 mètres à 6 mètres : 1 minute 30 secondes

Pour être plus proche des conditions réelles d'application, la décision d'application de la commission technique nationale de la FFESSM du 8/11/97 est observée :

→ vitesse de remontée de **15 mètres / minute,**

→ vitesse de remontée entre les paliers et du dernier palier à la surface de **1 mètre / 10 secondes.**

Temps Total de la plongée I : 55' + 1'30" + 2' + 30" + 36' + 30" = **95 minutes 30 secondes** soit ~ **1 heure 36 minutes**

Précautions particulières :

Compte tenu de l'existence d'azote résiduel dans les tissus des sujets à l'issue de la *Plongée Air*, il sera recommandé aux sujets de ne pas plonger ou de ne pas s'exposer à une ambiance hyperbare pendant les vingt-quatre heures qui suivent la compression. Après ce délai, il est classiquement admis que tout l'azote résiduel soit éliminé (cf.annexe 4).

➢ <u>Plongée au Nitrox *(Plongée Nitrox)* :</u>

55 minutes à 28 mètres (3.8 bars) ; gaz respiré = Nitrox ; plongée de type "carrée ".

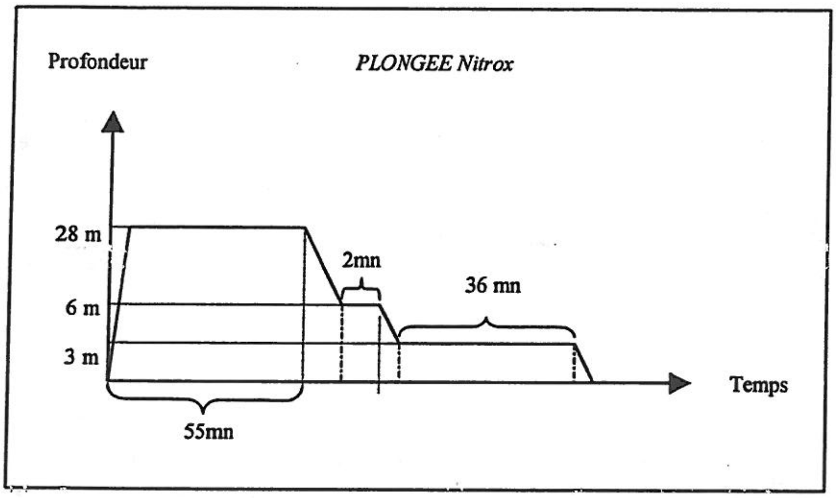

Les paramètres de cette plongée sont les mêmes que ceux de la *PLONGEE Air* ; seule variable : le gaz respiré n'est plus de l'Air Comprimé mais du Nitrox.

Temps de compression : identique au précédent → 3 minutes, qui seront comprises dans les 55 minutes pour calculer le PD.

Profil de Décompression : identique à celui de la *Plongée Air*

Choix du Nitrox : 36/64 → La pression partielle d'oxygène (PpO2) maximale rencontrée à 28 mètres sera de **1.368 bars** ; cette valeur est bien en deçà des **1.7 bars** théoriques et juste inférieure à la valeur standard recommandée par la plupart des organismes de plongée nationaux et internationaux qui est de **1.4 bars** (1, 5, 6, 19, 21).

NB : Comme lors d'une plongée Nitrox loisir, le mélange respiré aux paliers est le même que celui qui a été respiré tout au long de la plongée, en l'occurrence le Nitrox 36/64.

Précautions particulières :

Quand l'oxygène est utilisé en plongée, le principal souci est d'éviter la toxicité neurologique et accessoirement la toxicité pulmonaire pour des expositions très longues. La toxicité neurologique dépend de la **PpO2** à laquelle est exposé le plongeur et de la **durée d'exposition.**

Qu'en est-il pour la plongée que nous envisageons de réaliser ?

➔ Comme nous l'avons précédemment dit, nous exposerons nos sujets à une **PpO2 de 1.368 bars** ; valeur réputée inoffensive en plongée. Il faut aussi savoir que l'incidence générale des crises hyperoxiques est toujours plus basse en caisson hyperbare (où les facteurs favorisants de survenue de la crise hyperoxique comme le froid, l'hyperthermie, la fatigue ou le stress sont moins présents) (4). Le risque potentiel pour nos sujets est donc très faible.

➔ Concernant le temps d'exposition, les durées d'exposition maximales de plongée habituellement observées pour chaque PpO2 sont celles données par la National Oceanic and Atmospheric Administration (NOAA). Ces valeurs sont notées en annexe 2. On peut lire que la durée d'exposition maximale préconisée à 1.4 bars par la NOAA pour une plongée par jour est de **150 minutes** ➔ la durée totale de la *Plongée Nitrox* est de **95 minutes et 30 secondes.**

➔ Concernant maintenant la toxicité pulmonaire d'une plongée, elle peut être évaluée par le calcul de l'unité de référence : l'Unit Pulmonary Toxic Dose (UPTD) (12). La formule qui permet donc d'évaluer cette toxicité est la suivante, elle prend en compte la PpO2 maximale (1.368 bars) et la durée maximale d'exposition (96 minutes) :

$$\text{UPTD} : 96 \times (0.5 / [1.368-0.5])^{-0.833} = \mathbf{152}$$

NB : le seuil de toxicité est à 615 UPTD.

➔ Enfin, l'autre façon classique d'évaluer la toxicité due à l'oxygène est de calculer les OTU (Oxygen Toxicity Unit). Cette unité de toxicité de l'oxygène mise au point en 89 par l'hyperbariste Hamilton (7), quantifie les deux types de toxicité et permet en outre des cumuls de valeurs sur plusieurs jours. Elle est la valeur de référence pour de nombreux fabricants d'ordinateurs de plongée. Le tableau présenté en annexe 3 permet de calculer les OTU pour la *Plongée Nitrox*: **156.5** ➔ le seuil de toxicité journalière est de **850 OTU**.

Ces quelques notions indiquent :

- **que la *Plongée Nitrox* est tout à fait proche d'une plongée loisir habituelle en terme d'exposition à l'oxygène,**
- **et qu'elle se situe en deçà des seuils de toxicité admis.**

Elles doivent aussi être connues afin de gérer une éventuelle oxygénothérapie qui s'imposerait si un ADD survenait à l'issue de la *Plongée Nitrox*.

- Bien que le mélange respiré au cours de cette plongée soit suroxygéné et que l'azote résiduel soit théoriquement inférieur, comme pour la *Plongée Air*, nous recommanderons d'attendre 24 heures avant de replonger.

24- Etude Echodoppler

Un échodoppler cardiaque sera réalisé par le Dr Asfar (cardiologue de formation) chez tous les sujets à la sortie du caisson (T0) puis à T30, T60 et T90. Compte tenu des paramètres des plongées réalisées, le pic bullaire devrait apparaître au cours de la première heure après le retour en surface et devrait donc être enregistré. L'échocardiographie permet de localiser exactement la « fenêtre doppler » sur l'infundibulum pulmonaire du ventricule droit et de visualiser les bulles circulantes. Le doppler permet une meilleure détection (recrute plus de « bulleur ») et la « ventilation » des sujets bulleurs en différents grades est plus précise. Les différents résultats seront enregistrés sur bandes magnétiques vidéo pour une analyse ultérieure (cinq minutes à chaque enregistrement). La détection se fera d'abord sur le sujet en position allongée au repos puis après trois flexions extensions des membres inférieurs afin de mobiliser d'autres bulles. L'intensité du débit bullaire s'évaluera grâce à la cotation établie par Boussuges (2). Elle intègre la cotation doppler de Spencer et la cotation échocardiographique de Powell (18). Le débit bullaire est ainsi quantifié en cinq grades allant de 0 à 4 :

Grade 0 : absence de bulles à l'échocardiographie et au doppler

Grade 1 : détection de quelques bulles à l'échodoppler mais la grande majorité des cycles cardiaques n'en véhiculent pas.

Grade 2 : L'échocardiographie détecte un flot de bulles ; le doppler permet de préciser que moins de la moitié des cycles cardiaques véhiculent des bulles.

Grade 3 : L'échocardiographie détecte un flot de bulles ; le doppler permet de préciser que plus de la moitié des cycles cardiaques véhiculent des bulles.

Grade 4 : Le flot de bulles détectées à l'échocardiographie remplit les cavités cardiaques ; le doppler permet de préciser que tous les cycles cardiaques véhiculent des bulles.

Comme il a été précisé plus haut, un effort standardisé sera demandé à chaque sujet au cours de la plongée afin que les activités musculaire, cardiaque et vasculaire soient comparables à celles d'une plongée loisir classique. Nous essayons ainsi d'assurer des conditions de genèse du phénomène bulleux (saturation tissulaire, activité musculaire, turbulences hémodynamiques et phénomène de cavitation) aussi proches que possible de la réalité.

NB : Comme les sujets ne feront pas les deux plongées le même jour et afin que les phénomènes bulleux soient comparables d'un jour à l'autre pour un même sujet, il sera demandé à tous les sujets d'observer une activité équivalente (charge de travail, temps de repos, alimentation, absence de plongée, absence de respiration d'oxygène normobare ou hyperbare, absence de séjour en altitude ou de voyage en avion) la veille et le jour de l'expérimentation. Ces précisions seront notées dans le « Formulaire d'information ».

➢ **Paramètres de jugement**

- Critère de jugement principal :

Chaque mesure échodoppler est cotée de 0 à 4 ; chaque mesure est réalisée au repos et à l'effort à 4 temps différents.

Pour chaque sujet, une moyenne des 4 mesures effectuées à l'effort et une moyenne des 4 mesures effectuées au repos seront calculées. Par ailleurs afin de tenir compte de l'intensité du phénomène bullaire au cours du temps nous calculerons l'indice de sévérité de Kisman (11) et nous comparerons ainsi cet indice obtenu à l'issue des deux plongées (Mann et Withney pour les comparaisons intergroupes et Wilcoxon pour les comparaisons intra-groupes).

Exemple: le plongeur n présente au repos à l'issue de la plongée à l'air comprimé et à l'issue de la plongée au Nitrox les grades suivants:

	T 0	T 30	T 60	T 90
Pl AC	1	1	3	3
Pl Nx	2	2	2	2

Si la somme des grades est faite à l'issue des deux plongées, nous obtenons 8 pour les deux plongées. Cette somme évalue de façon globale l'intensité du phénomène bullaire sur 90 minutes dans les deux cas mais ne rend pas compte du fait qu'à l'issue de la plongée à l'air comprimé, le phénomène bulleux s'est intensifié à T60 et T90 (avec une augmentation présumée du risque d'ADD) alors que le phénomène bulleux est resté stable (et à un niveau présumé plus sûr) à l'issue de la plongée au Nitrox.

Nous proposons dés lors d'utiliser l'indice de sévérité de Kisman qui rendra compte de cette différence:
La formule de l'Indice de sévérité sous sa forme simplifiée est

$$S(\alpha) = [\ 1/\ 0.02.4^{\alpha}.(t90 - t0)\ ] \times [\ (d30^{\alpha} + d0^{\alpha})x(t30 - t0) + (d60^{\alpha} + d30^{\alpha})x(t60 - t30) + (d90^{\alpha} + d60^{\alpha})x(t90 - t60)]$$

où t est le temps de la mesure

d est le grade de la mesure échodoppler réalisée au temps t

α est l'exposant du degré bullaire, il prend en compte le fait que le grade bullaire n'évolue pas de façon linéaire à la quantité de bulles et au risque d'ADD,

S varie de 0 à 100.

Pour notre exemple S(AC) = 21.6 et S(Nx) = 12.5 ➔ l'indice de sévérité distingue donc la "sévérité particulière à chaque décompression" dont rendent compte les deux séries de mesures

- Critère de jugement secondaire :

Fatigue éprouvée par le plongeur cotée de 1 à 5 et relevée à T0, T30 et T60 et T90.

24- Incidents au cours de l'étude

- En cas de douleur tympanique ou sinusienne au cours de la compression, celle-ci sera ralentie ou arrêtée. Si ces mesures ne suffisent pas, la pression pourra être temporairement diminuée. Si cette dernière mesure ne suffit pas, le plongeur sera évacué pour être ramené à la pression atmosphérique.
  Un examen otoscopique avant l'entrée dans le caisson permettra de s'affranchir de l'existence d'une otite.

- En cas de crise convulsive à l'occasion d'une plongée Nitrox, le sujet sera remis en ambiance AC et sera conditionné pour assurer la libération des voies aériennes et recevoir une injection intraveineuse d'un milligramme de Rivotril. Après avoir recouvert la conscience, il sera évacué avec le médecin accompagnant.

- En cas de survenue de signes précurseurs d'ADD à l'issue d'une des plongées (asthénie intense, prurit, douleur articulaire, signes fonctionnels évoquant une anomalie de l'examen neurologique), une administration d'oxygène normobare et une réhydratation adaptée à l'état clinique du patient sera débutée en attendant une recompression thérapeutique en caisson selon le protocole en vigueur dans le service (oxygénothérapie normobare pendant 30 minutes puis surveillance pour le prurit, 2.5 ATA pendant 90 minutes sous FiO2 100% pendant 90 minutes pour les ADD de type I et tables 6 et 6A de l'US Navy pour les ADD de type II et la surpression pulmonaire) si son état clinique le justifie.

III) ANALYSE STATISTIQUE

Après avis du Dr Bruno Vielle (service de biostatistique), l'analyse statistique sera menée comme suit :

- Les valeurs seront exprimées en moyenne ± déviation standard.

- Pour détecter une chute de 1/3 du phénomène bulleux dans une population composée à part égale de "forts bulleurs" (indice de Kisman :16 ± 8) et de "moyens bulleurs" (4 ± 1.5) [16] avec une puissance de 80% et un seuil de significativité de 5%, un effectif de **18 sujets** est nécessaire pour mettre en évidence une diminution du phénomène bulleux de 30% entre les plongées pratiquées à l'AC et au Nitrox.

- Pour les comparaisons entre les deux groupes, on utilisera le test non paramétrique de Kruskall Wallis.

- Pour les comparaisons entre les deux plongées réalisées par un même plongeur, on utilisera le test non paramétrique de Wilcoxon.

- Une valeur de $p < 0.05$ sera considérée comme significative.

IV) ANNEXES

Annexe 1

Tables Marine Nationale 90, référence pour la FFESSM → TSVP

Annexe 2

Même pour des PpO2 inférieures à 1.7 bars, il existe toujours une durée d'exposition à « l'oxygène hyperbare » au-delà de laquelle le risque de convulsion est présent pour une majorité de plongeur. Ainsi, il a été déterminé (de façon empirique) pour les PpO2 usuelles une **durée maximale de plongée** à ne pas dépasser. Chaque marine dispose donc d'abaques (établies en fonction de leur propre expérience) précisant ces valeurs. Celle qui suit a été établie par la National Oceanic and Atmospheric Administration (NOAA). Elle est souvent considérée comme la plus sûre. Elle présente pour plusieurs PpO2 la durée maximale d'une plongée unique par jour et la durée cumulée de plusieurs plongées successives réalisées dans une même journée.

PpO2	Durée maximale pour 1 plongée/jour	Durée maximale pour plusieurs plongées/jour
0.6 bar	720 minutes	720minutes
0.7	570	570
0.8	450	450
0.9	360	360
1	300	300
1.1	240	270
1.2	210	240
1.3	180	210
1.4	150	180
1.5	120	180
1.6	45	150

Annexe 3

Table OTU

En fonction de la pression partielle d'oxygène et du temps passé en profondeur

PpO2 (en bars)	OTU / minute
0.5	0
0.6	0.27
0.7	0.47
0.8	0.65
0.9	0.83
1.0	1.00
1.1	1.16
1.2	1.32
1.3	1.48
1.4	1.63
1.5	1.78
1.6	1.92

d'après R.W. Hamilton de Hamilton Research LTD.

Annexe 4

D'après les mesures de sécurité préconisées par la FFESSM, tout plongeur loisir ayant respiré de l'air comprimé devra attendre douze heures avant d'éliminer l'azote résiduel. A l'issue de ce délai, il est classiquement admis que le tissu possédant la période la plus longue (120 minutes) ait perdu tout l'azote accumulé au cours de la plongée et qu'une nouvelle plongée peut être réalisée comme si elle était la première. En deçà de ce délai, le plongeur serait obligé de majorer ses paliers pour tenir compte de l'azote résiduel (20).

En demandant à nos sujets d'attendre vingt-quatre heures avant de replonger ou de s'exposer à une ambiance hyperbare, la marge de sécurité est majorée

V) BIBLIOGRAPHIE

1- Blanchard JL, Kersalé JY, « Manuel de plongée au Nitrox », FFESSM – Commision Technique Nationale.

2- Boussuges A, Carturan D, Ambrosi P, Habib G, Sainty JM, Luccioni R ; Decompression induced venous gas emboli in sport diving: Detection with 2D echocardiography and pulsed Doppler ; Int. J. Sports Med. 19 (1998) 7 –11.

3- Carturan D, Boussuges A, Burnet H, Fondarai J, Vanuxem P, Gardette B. Circulating venous bubbles in recreationnal diving : relationship with age, weight, maximal oxygen uptake and body fat pourcentage. Int J Sports Med 1999 ; 20 :410-414

4- Donald K. Oxygen and the diver. Worcester, England :The SPA Ltd, 1992. (actuellement disponible à la British Library).

5- Edmonds carl. Technical Diving. SPUMS Journal Vol 27 N°3, September 1997.

6- Elliot DH, Advanced recreationaal diving, some hazards of nitrox and technical diving.

7- Hamilton RW, Tolerating exposures to high oxygens levels ; Repex and others Methods ; Marine technology Society, 1989.

8- Hamilton RW. Does EAN improve decompression safety on no-stop dives ? Aquacorps Journal, 1995, vol 11 :21-22

9- Hugon M, Aptitude médicale à la plongée aux mélanges suroxygénés. In Bulletin de Médecine Subaquatique et Hyperbare, Plongées et Nouvelles technologies, 1997, Tome 7, Supplément.

10- Juvenspan Henri, Thomas Christian ; "Plonger aux mélanges - Plongées profondes, utilisation de l'oxygène", Ed Ulmer

11- Kisman K, Masurel G. Evaluation de la qualité d'une décompression basée sur la détection ultrasonore de bulles, Med. Aero. Spat., Med. Sub. Hyp., tome 17, n°67, 1978, p.293-297.

12- Lambertsen CJ, « Practical aspects of oxygen tolerance and oxygen toxicity, oxygen tolerance curves, precaution against oxygen toxicity. Quantification of unit pulmonary toxic dose (UPTD). » Report Institute for Environm. Med., University of Pennsylvania, 1970.

13- Lanphier EH, « Man in pressure », In : Handbook of physiology, Adaptation of the environment, Am. Physiol. Soc, Washington DC, 1964, chapitre 58, 893-909.

14- Lanphier E.H. 1955. Nitrogen-oxygen mixture physiology. Phases 1 and 2. U.S. Navy Experimental Diving Unit, Washington, Report 7-55.

15- Lanphier E.H. 1958. Nitrogen-oxygen mixture physiology. Phases 4 and 6. U.S. Navy Experimental Diving Unit, Washington, Report 7-58.

16- Nishi RY, Kisman KE, Eatock BC, Buckingham IP, Masurel G ; « Assessment of decompression profiles and divers by doppler ultrasonic monitoring », Proceeding of the 7th symposium of underwater physiology, 1981,vol 7, pages 717-727.

17- Nishi RY. Doppler and ultrasonic bubble detection . Bennet et Elliot, The physiology and medecine of diving. Chap 15,pages 432-453.Edition 92.

18- Powell MR, Spencer MP, Von Ram O ; Ultrasonic surveillance of decompression in « The physiology and medecine of diving », Bennett and Elliott, Chapitre 16, pages 404-434. Edition 1982.

19- Sirven D, « La plongée Nitrox, Guide d'utilisation des mélanges Nitrox pour les plongeurs loisirs », manuel produit pour l'organisme de plongée américain Technical Diving International.

20- Trucco JN, Biard J, Redureau JY, Fauvel Y ; « Tables Marine Nationale 90 » version du 3/5/1999, Comité interrégional Bretagne et Pays de la Loire, Commission Technique Régionale.

21- Verdier Cedric - « La plongée Nitrox pour tous » - SAGA, collection Pédagogie.

VI) PUBLICATIONS ET COMMUNICATIONS DE L'EQUIPE CONCERNANT L'HYPERBARIE

- GOUELLO JP, BOUACHOUR G, PERSON B, RONCERAY J, CELLIER P, ALQUIER Ph.
  Intérêts de l'oxygénothérapie hyperbare dans la pathologie digestive post-radique.
  36 observations.
  Presse Med 1999, 28, 20, 1053-1057.

- MATHIEU D, WATTEL F, MATHIEU-NOLF M , DURAK B, TEMPE JP, BOUACHOUR G, SAINTY JM.
  Etude prospective randomisée multicentrique comparant l'efficacité de l'oxygénothérapie hyperbare à 12 heures d'oxygénothérapie normobare dans les formes non comateuses d'intoxication au CO. Résultats de l'analyse intermédiaire.
  XXVème congrès de la société de réanimation de langue française
  Paris 22-24 janvier 1997 (poster)
  Rean. Urg. 1996, 5, 6, 805 (abstract).

- BOUACHOUR G, GOUELLO JP, ALQUIER Ph.
  L'oxygénothérapie hyperbare : les indications en urgence.
  Rev. Prat. Médecine Générale 1994, 8, 246, 21.

- NEVIERE R, MATHIEU D, MATHIEU-NOLF M, TEMPE JP, BOUACHOUR G, SAINTY JM, GRANDJEAN B, WATTEL F.
  HBO versus NBO in the treatment of minor carbon monoxide intoxications : preliminary results of a french multicentric double-blind and randomized study.
  First European Consensus Conference on Hyperbaric Medecine
  Lille, 19-20 septembre 1994.

- DUBOIS R, GOUELLO JP, GUIRAUD MP, BOUACHOUR G, DUBIN J.
  Cellulites cervicales : apports de l'oxygénothérapie, à propos de 12 cas.
  Journées Multirégionales d'ORL
  Lyon, 28-30 avril 1994.

- GOUELLO JP, BOUACHOUR G, GAGNEUX C, ALQUIER Ph.
  Variations humorales du facteur atrial natriurétique et du système rénine angiotensine aldostérone en hyperbarie chez le sujet normal.
  Bul. Med. Sub. Et Hyp. 1995, 55, 65-71.

- BOUACHOUR G, CRONIER P, GOUELLO JP, TOULEMONDE JL, TALHA A.
  Hyperbaric oxygen therapy in the management of crush injuries : a randomised double-blind placebo controled clinical trials.
  The Journal of Trauma : injury, infection and critical care ; 1996, 41, 2, 333-339.

VII) BESOINS FINANCIERS

Le service de Réanimation Médicale met son matériel (caisson hyperbare, respirateurs, échographe et ergocycle) à la disposition des investigateurs.

Le Nitrox 36/64 sera obtenu grâce au paramétrage des respirateurs.

Aucun matériel consommable et aucun examen complémentaire ne seront nécessaires.

Les médecins investigateurs et accompagnant sont bénévoles.

Assurances.

Droit du CCPPRB.

VIII) ENGAGEMENT DES INVESTIGATEURS

- Les « plongées » se dérouleront en fin d'après-midi afin de ne pas perturber les séances quotidiennes d'oxygénothérapie hyperbare prévue dans le service et de faciliter la participation des bénévoles.

- Si l'indication d'une séance urgente d'oxygénothérapie hyperbare était posée pour un patient de l'hôpital, la « plongée » en cours serait immédiatement interrompue afin de libérer un compartiment du caisson pour l'urgence.
