# Supplementary material for: Enriched Air Nitrox Breathing Reduces Venous Gas Bubbles after Simulated SCUBA Diving: A Double-Blind Cross-Over Randomized Trial
Source: PLoS One. 2016 May 10;11(5):e0154761. doi: 10.1371/journal.pone.0154761 (PMC4862661; doi:10.1371/journal.pone.0154761)
Supplement: S3 File — (DOC) [file pone.0154761.s003.doc]

**Enriched air nitrox breathing reduces venous gas bubbles after simulated SCUBA diving: a double-blind cross-over randomized trial**

**Summary of study protocol**

- The study was approved by the local Human Subjects committee.
- Written informed consent was obtained from all volunteers.
- The current investigation was a single center, prospective, double-blind study with crossover design.
- Inclusion criteria: over 18 years of age, diving experiences: French recreational diver license and absence of contraindication to dive.
- Exclusion criteria: history of decompression accident(s), oxygen administration or diving within 24 hours before simulated dive, or absent or low intravascular bubble production (Bubble score ≤ 1) after initial test decompression.
- Simulated dives took place in a hyperbaric chamber.
- 47 divers underwent a simulated dive in order to exclude subjects with low intravascular bubble production.
- Out of the 47 volunteers, 12 divers were selected to undergo a subsequent simulated dives in a randomized, double-blind, crossover setup.
- Subjects were randomized between breathing air (FiO_2_ 0.21) or enriched air nitrox (EAN; FiO_2_ 0.36) throughout the hyperbaric protocol.
- Subjects breathed through a mouthpiece connected to a mechanical ventilator (Servo 900C, Siemens, Sweden), with a pressure support of 10 cmH_2_O.
- Subjects were accompanied by a physician in the hyperbaric chamber, both blinded to FiO_2_.
- The diving profile was designed according to the decompression table of the French Navy and was used for all simulated dives in our study, see attached figure.
- Subjects cycled at an energy expenditure of 50W at 4 episodes of 5 minutes during bottom time on a stationary bicycle placed in the HC, in order to simulate energy expenditure during diving.
- Temperature in the HC was maintained at 22-25 ̊ Celsius during stabilization of depths.
- Intravascular bubbles were assessed by pulsed Doppler measurements of the trunk of the pulmonary artery by a certified cardiologist, at 0, 30, 60 and 90 minutes after decompression.
- Images were recorded and analyzed offline by two independent reviewers.
- Bubble scores were based on the previously described score system of Spencer.
